# Dissociable effects of acute SSRI (escitalopram) on executive, learning and emotional functions in healthy humans

**DOI:** 10.1038/s41386-018-0229-z

**Published:** 2018-09-26

**Authors:** Nikolina Skandali, James B. Rowe, Valerie Voon, Julia B. Deakin, Rudolf N. Cardinal, Francesca Cormack, Luca Passamonti, William R. Bevan-Jones, Ralf Regenthal, Samuel R. Chamberlain, Trevor W. Robbins, Barbara J. Sahakian

**Affiliations:** 1 Department of Psychiatry, University of Cambridge, Cambridge, UK; 2 Medical Research Council/Wellcome Trust Behavioural and Clinical Neuroscience Institute, University of Cambridge, Cambridge, UK; 3 Cambridgeshire and Peterborough NHS Foundation Trust, Cambridge, UK; 4 Department of Clinical Neurosciences, University of Cambridge, Cambridge, UK; 5 Cambridge Cognition, Cambridge, UK; 6 Division of Clinical Pharmacology, Rudolf-Boehm-Institute of Pharmacology and Toxicology, Leipzig University, Medical Faculty, Leipzig, Germany; 7 Department of Psychology, University of Cambridge, Cambridge, UK

## Abstract

Serotonin is implicated in multiple executive functions including goal-directed learning, cognitive flexibility, response inhibition and emotional regulation. These functions are impaired in several psychiatric disorders, such as depression and obsessive–compulsive disorder. We tested the cognitive effects of the selective serotonin reuptake inhibitor escitalopram, using an acute and clinically relevant dose (20 mg), in 66 healthy male and female volunteers in a double-blind, placebo-controlled study. Participants performed a cognitive test battery including a probabilistic and reversal learning task, the CANTAB intra-dimensional/extra-dimensional shift test of cognitive flexibility, a response inhibition task with interleaved stop-signal and No-Go trials and tasks measuring emotional processing. We showed that acute escitalopram administration impaired learning and cognitive flexibility, but improved the ability to inhibit responses in stop-signal trials while leaving unaffected acute emotional processing. Our findings suggest a dissociation of effects of acute escitalopram on cognitive functions, possibly mediated by differential modulation of brain serotonin levels in distinct functional neural circuits.

## Introduction

Serotonin (5-HT) is implicated in learning, executive and affective functions [1]. Much of the relevant evidence has depended on examining the effects of 5-HT loss in humans using acute dietary tryptophan depletion (ATD) [2], or in experimental animals, using 5-HT neurotoxins such as 5,7-dihydroxytryptamine (5,7-DHT) (e.g. [3]). ATD impairs visual discrimination learning and reversal [4], while selective 5-HT depletion in the amygdala has been shown to increase sensitivity to aversive feedback in probabilistic learning and reversal tasks [5]. This is analogous to the effects of an acute low dose of citalopram in healthy volunteers [6] and rats [7] hypothesised to arise from inhibitory effects on 5-HT transmission [8]. However, higher doses or sub-chronic treatment with citalopram improve performance in probabilistic reversal learning tasks in rats [7]. These findings have clinical implications, as depressed humans show deficits in cognitive flexibility [9] and exaggerated reactions to negative feedback mediated by the prefrontal cortex (PFC) and the amygdala [10]. Moreover, selective serotonin reuptake inhibitors (SSRIs) constitute the first-line treatment for mood disorders [11].

This study examined the effects of acute escitalopram in healthy human volunteers on a test battery used extensively in both human and animal studies, assessing fronto-executive functions and previously shown to be sensitive to serotonergic manipulations [6, 12]. We included (i) a probabilistic and reversal learning task; (ii) the CANTAB Intra-dimensional/Extra-dimensional set shift test, which includes reversal learning components; (iii) a combined Stop-signal/Go/No-Go response inhibition task that measures both restraint and cancellation types of response inhibition in the same subjects [13] and (iv) emotional processing and social cognition tasks including the CANTAB affective Go/No-Go task, and the face affective Go/No-Go task and social information preference task, both from the newly developed EMOTICOM test battery [14]. Detailed hypotheses are described in the Methods section. An additional important feature of this study was its relatively novel examination of possible influences of gender, depressive symptoms and trait anxiety on the effects of escitalopram.

## Materials and methods

### Participants

The study was jointly sponsored by the University of Cambridge and Cambridgeshire and Peterborough NHS Foundation Trust and approved by the NHS East of England—Cambridge Central Research Ethics Committee (REC reference: 15/EE/0004). All participants were provided with verbal and written information on the study and gave written consent. Exclusion criteria included current or past psychiatric symptoms using a clinical structured interview (MINI International Neuropsychiatric Interview; [15]), personal or family history of psychiatric and neurological diseases, significant active or past medical problems, alcohol or drug abuse and excessive nicotine consumption (details in Supplementary Materials and Methods). Blood samples were collected at 2.5 and 5.5 h after drug administration.

### Study design

A double-blind, parallel-groups design was employed to eliminate effects of extensive training in learning tasks [16]. We administered escitalopram 20 mg, equivalent to 40 mg of the racemic compound citalopram [17], as the activity of citalopram lies primarily on the S-enantiomer (escitalopram) and acute high dose of escitalopram was shown to alter electrophysiological measures of information processing [18].

### Mood state and personality trait questionnaires

Participants were assessed for changes in mood state and drug side effects by completing computerised visual analogue scales at three time points. Participants also completed well-validated mood state and personality trait questionnaires, including the Beck Depression Inventory (BDI) [19] and State-Trait Anxiety Inventory (STAI) [20].

### Neuropsychological testing

Neuropsychological testing started 3 h following pill administration to achieve peak plasma escitalopram concentrations [21]. Overall, 65 healthy volunteers completed the testing session (placebo; *N* = 33, escitalopram; *N* = 32) as one participant experienced side effects. Groups were matched demographically and in terms of baseline mood (Table 1).

**Table 1:** Group demographics

Measures	Placebo group	Escitalopram group	Group difference^a^
Male:female	17:16	16:16	*p* = 0.903
Age	25 (5)	27 (7)	*p* = 0.061
NART	42.45 (4.93)	42.9 (5.27)	*p* = 0.723
Years of education	16.6 (2.7)	16.9 (2.5)	*p* = 0.693
STAI trait anxiety score	35.3 (8.6)	34.6 (9.5)	*p* = 0.77
BDI score	5.3 (5.1)	4.1 (4.4)	*p* = 0.32

Mean (SD)

^a^Group difference: *p*-values of chi-square test for gender and two-tailed *t* tests for the other measures

### Statistical analysis

Data were analysed with SPSS software, version 23.0. Appropriate statistical tests were applied, including chi-square test, two-tailed *t* tests and ANOVAs, based on a priori hypotheses. Due to the number of tests administered, the Benjamini–Hochberg procedure was applied [22] for false discovery rate control (i.e. controlling the number of ‘false positives’ in our results) set a priori [23] at *q* < 0.15 [24]. The false discovery rate assumes positive dependence or independence among variables, as was the case in our data, and it was performed across all hypotheses tested to provide a strict control of the false discovery rate. The Benjamini and Hochberg corrected significance level was 0.018.

### Task description

1. Probabilistic reversal learning task [6]: Participants made a two-alternative forced choice between two patterns (green and red) over a series of trials by touching the screen. Feedback was presented on the screen after each choice as ‘CORRECT’ and ‘INCORRECT”. The pattern chosen in the first trial of the first stage was the correct one and participants received a ratio of 80:20 of accurate:misleading feedback for this in the first stage. Then the stimulus–outcome contingencies reversed (Figure S1 in Supplementary Materials and Methods).

Primary outcome measures, defined as per Chamberlain et al. [6], were the number of errors until reaching learning criterion (eight correct consecutive responses to the most rewarding stimulus) in Stage 1 (indicating ability to encode the stimulus–outcome contingencies) and Stage 2 (indicating ability to learn the new stimulus–outcome contingencies). Secondary outcome measures included probabilities of win (reward)-stay/lose (no-reward)-shift strategy for each stage defined as per Rygula et al. [5]. Based on Chamberlain et al. [6], we hypothesised that the escitalopram-treated group would perform more errors during the probabilistic learning and after reversal and would show higher sensitivity to misleading feedback.

2. CANTAB Intra-Extra dimensional set shift task [25]: This is a nine-stage task consisting of visual discrimination, attention set formation and rule acquisition, maintenance of attention, set-shifting and flexibility of attention and rule reversal. Primary measures [26] included errors in the critical stages of intra-dimensional set shift (IDS) (requiring the ability to apply a rule to new stimuli) and intra-dimensional shift reversal (IDR), extra-dimensional set shift (EDS) (requiring the ability to redirect attention towards a previously irrelevant dimension) and extra-dimensional shift reversal (EDR). Secondary measures included errors and response latencies in all stages.

3. Response inhibition task; the integrated Stop-signal and No-Go trials paradigm [27]: Participants were presented with three types of trials; 360 Go trials (75%) requiring a right or left button press depending on the direction of a black arrow on the screen, 80 Stop-signal trials (17%, with ~50% successful) requiring cancellation of a cued button press when the black arrow turns red and 40 No-Go trials (8%) requiring withholding themselves from pressing any button as the arrow appears red (Figure S2 in Supplementary Materials and Methods). Primary outcome measure was the estimated Stop-signal reaction time (SSRT) (i.e. the time required to abort an initiated action in the presence of a stop-signal) defined as per Ye et al. [27].

4. Emotional processing tasks: *EMOTICOM face affective Go/No-Go task* [14]: Participants are requested to press space bar in response to a specific target emotion when presented with blocks of facial stimuli (happy, sad and neutral facial expressions) as targets and distractors. Affective response bias was calculated as the difference in reaction time (RT) between the happy target/sad distractor condition and the sad target/happy distractor condition as per Bland et al. [14]. *CANTAB affective Go/No-Go task* [28]: Participants need to make a button press when they see a word of a specific valence (e.g. positive) in a series of blocks of two words with distinct emotional valence (positive, negative and neutral). Affective response bias was calculated as the difference in correct RTs between positive and negative blocks as per Murphy et al. [28]. *EMOTICOM social information preference (‘Theory of mind’) task* [14]: Participants are presented with socially ambiguous scenarios with pieces of information hidden from view, including three faces (revealing feelings), three thoughts and three facts. They select up to four pieces of information and then choose between three possible outcomes of neutral, positive or negative valence. Interpretational affective bias was calculated as the difference in the proportion of selected positive and negative scenario outcomes as per Bland et al. [14].

### Study hypotheses

We hypothesised, based on prior literature on serotonergic interventions (Table S1 in Supplementary Materials and Methods), that participants receiving an acute escitalopram dosage of 20 mg will make more errors during both probabilistic learning and after reversal of stimulus reward contingencies (perseverative errors) [6]. We predicted no effect or possible improvement [27] on tests of action restraint or cancellation, as previous studies applying Stop-signal paradigms produced mixed results and no behavioural effect on No-Go responding. Deficits were expected in tests of cognitive flexibility (although predominantly after reversal). We also hypothesised that acute escitalopram would influence affective bias in the three emotional processing tasks, as negative affective biases are well-documented in major depression [29] and following ATD in healthy volunteers [28], and acute administration of a clinically relevant dosage of citalopram in healthy volunteers was previously shown to increase recognition of happy faces and attention to socially relevant stimuli [30].

## Results

### Biochemical analysis (placebo; *N* = 29, escitalopram; *N* = 30)

Mean escitalopram plasma concentration at 2.5 h was 14 ng/ml (SD: 5.72, *p* < 0.001, t(54) = 18.835), and at 5.5 h was 17.24 ng/ml (SD: 4.27, *p* < 0.001, t(54) = 20.548).

1. Probabilistic reversal learning task (placebo; *N* = 33, escitalopram; *N* = 31).

*Stage 1 acquisition phase*: The learning criterion was achieved by 26/31 participants in the escitalopram group and 30/33 participants in the placebo group, *χ*^2^ = 1.393, *p* = 0.268. However, the escitalopram group made significantly more errors, *U* = 314, *z* = −2.850, *p* = 0.004 (Table 2).

**Table 2:** Mean (SD) errors and response latencies and probabilities of win–stay/lose–shift behaviour based on feedback type (80% valid, 20% misleading) in the two treatment groups

	Measure		Placebo	Escitalopram	Group difference^a^
Stage 1	Errors		1.76 (3.73)	4.55 (6.66)	***p*** = 0.004
	RTs (ms)		2016.06 (937.32)	2165.79 (968.95)	*p* = 0.532
	*Feedback type* 80% accurate 20% misleading				
Probabilities	Reward–stay	80%	86.74	75.6	*p* = 0.043
		20%	1.14	4.03	*p* = 0.104
	Reward–shift	80%	5.97	9.48	*p* = 0.125
		20%	4.92	8.47	*p* = 0.279
	Lose–stay	80%	1.42	4.13	*p* = 0.125
		20%	88.64	72.18	***p*** = 0.009
	Lose–shift	80%	5.87	10.79	*p* = 0.032
		20%	5.3	15.32	***p*** = 0.003
Stage 2	Errors		6.58 (2.57)	7.58 (4.46)	*p* = 0.689
	RTs (ms)		2251.86 (6285.86)	1280.32 (865.23)	*p* = 0.237
	*Feedback type* 80% accurate 20% misleading				
Probabilities	Reward–stay	80%	68.75	63.71	*p* = 0.376
		20%	11.74	15.32	*p* = 0.359
	Reward–shift	80%	6.06	8.57	*p* = 0.238
		20%	4.92	6.58	*p* = 0.442
	Lose–stay	80%	16.1	16.32	*p* = 0.237
		20%	70.08	62.9	*p* = 0.971
	Lose–shift	80%	10.07	12.6	*p* = 0.684
		20%	13.26	14.92	*p* = 0.355

^a^Group difference: *p*-values of one-way ANOVAs. Significant *p*-values following control of the false discovery rate of *q* < 0.15 with Benjamini–Hochberg procedure are shown in bold

*Stage 2 reversal phase*: The learning criterion was achieved by 25/31 participants in the escitalopram and 27/33 participants in the placebo groups, *χ*^2^ = 0.014, *p* = 0.904. Errors and RTs were both similar between groups, *p* > 0.05.

*Win-stay/lose-shift*: In Stage 1, a priori planned analyses showed that the escitalopram group had a significantly lower probability of win-stay, *p* = 0.043, F(1,62) = 4.272, partial *η*^2^ = 0.064, and a significantly higher probability of lose-shift, *p* = 0.013, F(1,62) = 6.575, partial *η*^2^ = 0.096. We further assessed sensitivity to valid (80% of trials) or misleading (20% of trials) feedback by separating the above probabilities based on feedback type. Table 2 shows that the escitalopram group had a significantly higher probability of lose-shift after both misleading, *p* = 0.003, F(1,62) = 9.724, partial *η*^2^ = 0.136, and accurate (valid) feedback, *p* = 0.032, F(1,62) = 4.834, partial *η*^2^ = 0.72.

The two groups did not differ in win-shift behaviour after accurate (valid) feedback, *p* > 0.05. There was no difference in either of these measures during Stage 2.

**Fig. 1:**
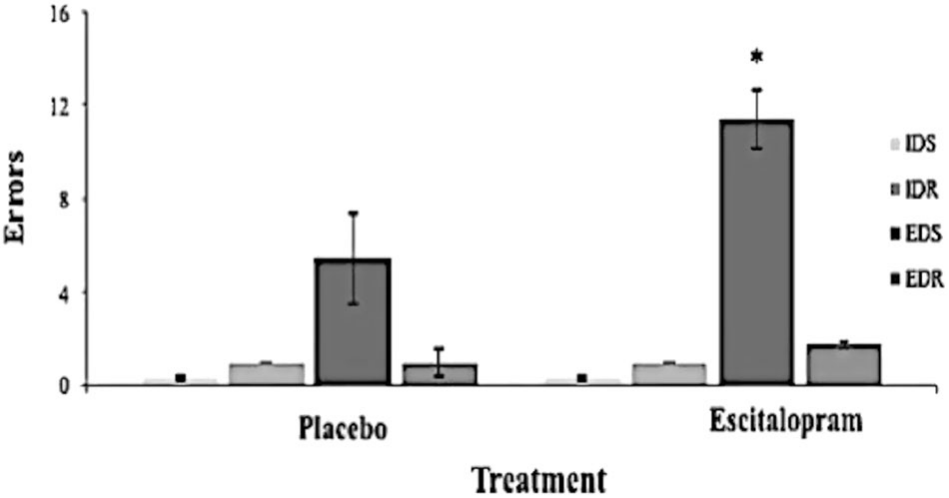
Escitalopram group made significantly more errors in the EDS, compared with the placebo group, following false discovery rate control of *q* < 0.15 with the Benjamini–Hochberg procedure, as denoted with an asterisk

2. CANTAB Intra-Extra dimensional (ID/ED) set shift task (placebo; *N* = 31, escitalopram; *N* = 30): Repeated measures ANOVA for errors in the four critical stages of IDS, IDR, EDS and EDR showed a significant stage by treatment effect, *p* = 0.026, F(3,52) = 3.339, partial *η*^2^ = 0.162. Both groups made fewer errors at the IDS compared with the EDS stage, *p* = 0.017, F(1,54) = 6.091, partial *η*^2^ = 0.101. Follow-up, one-way ANOVAs planned a priori for the IDS and EDS stages showed that the escitalopram group made significantly more errors at the EDS stage (mean: 11.4, SD: 10.49) than the placebo (mean: 5.43, SD: 6.93), *p* = 0.014, F(1,56) = 6.449, partial *η*^2^ = 0.103 (Fig. 1). Escitalopram did not affect IDS errors, *p* = 0.561. Follow-up, one-way ANOVAs for the reversal stages, IDR and EDR, also showed that escitalopram group made more errors at EDR, *p* = 0.082, F(1,59) = 3.135, partial *η*^2^ = 0.05, as shown in Fig. 1. The escitalopram group responded more slowly compared with the placebo in the EDS stage, *p* = 0.026, t(59) = −2.307, partial *η*^2^ = 0.102. There was no effect on errors or RTs in the learning stages 1-5, *p* > 0.05 (Table S2 in Supplementary Materials and Methods).

3. Response inhibition task (placebo; *N* = 33, escitalopram; *N* = 32): One participant outlier in the SSRT (>2 SD from group mean) was excluded. Table 3 shows that escitalopram significantly reduced SSRT (speeded response inhibition), *p* = 0.013, t(62) = −2.550, without significant changes in other measures.

**Table 3:** Groups significantly differ in SSRT, but not in other measures in the response inhibition task

Measures (mean + SD)	Placebo	Escitalopram	Group difference^a^
SSRT	198.82 (40.02)	175.77 (31.46)	***p*** = 0.013, t(62) = −2.550
Go reaction time	463.52 (127.21)	492.19 (123.65)	*p* = 0.364, t(62) = 0.914
Go omission error rate	0.005 (0.01)	0.01 (0.04)	*p* = 0.179, t(62) = 1.361
Go commission error rate	0.11 (0.07)	0.1 (0.07)	*p* = 0.621, t(62) = 0.498
No-Go error rate	0.11 (0.14)	0.08 (0.14)	*p* = 0.459, t(62) = 0.745

Variables represent RTs in milliseconds

^a^Group difference: *p*-value of independent samples *t* tests, significant results following false discovery rate control of *q* < 0.15 with the Benjamini–Hochberg procedure are denoted in bold

4. Effects of acute escitalopram on emotional processing: Table 4 shows that acute escitalopram administration did not alter affective bias in the three tasks, but did increase the proportion of facts chosen in the EMOTICOM social information preference task, *p* = 0.017, t = 2.451, partial *η*^2^ = 0.095, as shown with pre-planned independent samples *t* tests.

**Table 4:** Escitalopram effects on emotional processing tasks

Task		Escitalopram	Placebo	Group difference^a^
*CANTAB affective Go/No-Go task*				
Stimulus type	Words			
Target emotions	Positive, negative, neutral			
Sample size	*N* = 60 (placebo; 31, escitalopram; 29)			
**Measure**	Omission errors (shift blocks)	2.9 (3.89)	2.03 (2.4)	*p* = 0.843
	Omission errors (non-shift blocks)	2.62 (3.8)	1.87 (2.29)	*p* = 0.712
	Affective bias (in milliseconds)	−9.22	−1.15	*p* = 0.482
*EMOTICOM affective Go/No-Go task*				
Stimulus type	Faces			
Target emotions	Happy, sad, neutral			
Sample size	*N* = 64 (placebo; 32, escitalopram; 32)			
**Measure**	**Percentage of correct ‘hit’ responses for target emotion**			
	Target: happy, distractor: sad, condition	92.5%	88.1%	*p* = 0.470
	Target: happy, distractor: sad, condition	88.8%	88.1%	*p* = 0.906
	**Affective bias RT** (in milliseconds)	−0.013	−0.012	*p* = 0.896
*EMOTICOM social information preference task* (*‘Theory of mind’*)				
Stimulus type	Outcomes of socially ambiguous situations			
Target emotions	Positive, negative, neutral			
Sample size	*N* = 58 (placebo; 32, escitalopram; 27)			
Measure	**Affective bias in scenario outcome choices** (mean value)	2.78	1.91	*p* = 0.235
Stimulus type	Information type (faces, thoughts, facts)			
	**Proportion of facts** (mean ± SD)	0.15 (0.1)	0.1 (0.07)	***p*** = 0.017
	**Proportion of thoughts** (mean ± SD)	0.27 (0.11)	0.29 (0.1)	*p* = 0.330
	**Proportion of faces** (mean ± SD)	0.58 (0.12)	0.61 (0.1)	*p* = 0.346

Acute escitalopram administration increased the proportion of selected facts in the EMOTICOM Social information preference task following false discovery rate control at *q* < 0.15 with the Benjamini–Hochberg procedure, as denoted in bold, while leaving unaffected other measures in the emotional processing tasks. Mean (SD) for measures unless otherwise specified

^a^Group difference: *p*-values of independent sample *t* tests

### Subjective measures and effects of trait anxiety, BDI and sex

We found no interaction between treatment, and trait anxiety, BDI or sex in any task measure (*p* > 0.05 for all). Escitalopram had no significant effects on most subjective mood ratings (*p* > 0.05) (Table S3 in Supplementary Materials and Methods). The drug did enhance self-reported excitement (*p* = 0.028, F(1,29) = 5.368, partial *η*^2^ = 0.156, *p* > 0.05), but this was no longer present by the end of the testing session and did not contribute to any of the observed effects on the cognitive tests, following its inclusion as a covariate.

Variations in plasma escitalopram levels within the escitalopram group did not predict treatment effects on any of the aforementioned task measures (*p* > 0.05 for all).

## Discussion

This study shows reliable effects of an acute oral dose of the serotonin reuptake inhibitor escitalopram on cognitive as well as emotional functioning in healthy volunteers. Acute escitalopram impaired learning with uncertain reinforcement and enhanced responsivity to misleading negative feedback, analogous to that observed in depression. It produced dissociable effects on different aspects of executive function, specifically improving inhibitory response control (shorter SSRT), but impairing cognitive flexibility (impaired EDS performance). These differences reflect dissociable effects on functionally distinct aspects of executive function [31], leaving unaffected emotional processing at the group level, in healthy participants. These novel findings of acute effects of serotonin reuptake inhibition on cognitive performance have implications for understanding how central 5-HT pathways modulate cognition in health and psychiatric disorders.

### Effects on probabilistic and deterministic learning

Healthy adults on escitalopram made more errors to criterion during Stage 1 of the probabilistic learning task, similar to effects of citalopram [6]. Further analysis revealed that escitalopram increased lose-shifting after misleading negative feedback, a significant finding after controlling for false discovery rate. In other words, there was a detrimental effect reminiscent of patients with depressive disorders [10] and effects in rats treated acutely with low doses of citalopram or forebrain 5-HT depletion [7]. Escitalopram increased lose-shifting after accurate feedback, but this finding did not survive significance when controlling for false discovery rate. There was no overall tendency to shift regardless of reinforcing feedback, as win-shift responding was not changed. These effects contrast with those reported for this task using ATD by Murphy et al. [28].

At the early stages of learning in the CANTAB ID/ED shifting paradigm, there were no effects of escitalopram, contrasting with effects of ATD (e.g. [12, 32]). The relative lack of sensitivity of the latter visual discrimination-learning task may relate to its deterministic nature, whereas the probabilistic learning task entails greater uncertainty.

### Effects on inhibition and attention control

Acute escitalopram speeded SSRT. This cannot simply be attributed to strategic changes in responding as Go RT and other measures were unchanged. ATD has no significant effect on SSRT performance [33], although ATD can speed responding on punished Go trials of a Go/No-Go paradigm [34]. Whilst sharing many neurobehavioral processes of inhibitory response control, including the engagement of fronto-striatal ‘loops’ involving the right inferior frontal gyrus (BA 44 and 45) [35, 36], the Stop-signal and Go/No-Go paradigms appear to have some distinct underlying mechanisms [13]. Blockade of 5-HT reuptake with citalopram produced no effect on SSRT over a range of doses in rats [13, 37]. In humans, citalopram (30 mg) administration in healthy volunteers similarly produced no effect in a Stop-signal paradigm [6], although Ye et al. [27] showed that it improves SSRT and No-Go errors in Parkinson’s disease patients with relatively severe symptoms.

The effects of serotonin on behavioural inhibition depend on the paradigm employed and action restraint differs from action cancellation [38, 39]. The SSRT might reflect a more sensitive (behavioural) measure than Go/No-Go performance—indeed the study by Macoveanu et al. [40] failed to show any significant effects on behavioural measures (errors and reaction times) of intravenous (i.v.) citalopram (20 mg/h) on Go/No-Go or on a variant where an alternative response had to be made. Additionally, although Del-Ben et al. [39] showed enhanced activation of the lateral orbito-frontal cortex following citalopram i.v. 7.5 mg administration, no behavioural effect was observed. The present study is the first to show improvements in inhibitory response control performance by an acute high-dosage SSRI in healthy volunteers. The effects of serotonin manipulations may also vary according to baseline characteristics. For example, the change in activation of right inferior frontal gyrus during No-Go responding following ATD depends on neocortical 5-HT_2A_ receptor binding [40]. However, none of the effects of escitalopram on inhibition in the current study were affected by trait anxiety, depression, impulsiveness or gender.

Acute escitalopram treatment in healthy adults impaired extra-dimensional shift performance. Studies on rats (e.g. [41, 42]) and marmoset monkeys (e.g. [43]) implicate 5-HT in deterministic reversal learning deficits, rather than ED-shifting, with ATD impairing reversal learning (errors) and lengthening response latencies at certain stages of reversal and ID-shifting [32]. However, the common deficits produced on probabilistic learning and ED-shifting here suggest effects of the drug on neural circuits including the right ventro-lateral PFC (BA47), as both of these tasks are associated with activations of this region [44]. Moreover, the pharmacological fMRI study of Del-Ben et al. [39] emphasised strong interactions between i.v. administration of the SSRI citalopram and different task-related activations of this region.

### Effect on emotional processing

In contrast to its effects on learning and ‘cold’ executive performance, acute escitalopram had relatively little effect on emotional processing or so-called ‘hot’ cognition [45] (see Table 4), even taking into account trait anxiety, BDI and gender influences. Face recognition was measured in the EMOTICOM faces affective Go/No-Go task where participants had to respond to a target face ignoring the distractor, but Table 4 shows that escitalopram produced no effects on accurate ‘hit’ responses. The groups also did not differ in omission errors for distinct target valence words in the CANTAB affective Go/No-Go test-a task previously shown to be sensitive to ATD (enhancing negative affective bias) [29] and depression [28]. We did find a significant preference for subjects treated with escitalopram to use facts in preference to faces or thoughts to interpret ambiguous scenarios in the novel “Social Information Processing” task. This result suggests that informational aspects of socio-emotional processing used for decision-making may be diminished.

In relation to the published literature, Del-Ben et al. [39] found no behavioural effects on face recognition of i.v. citalopram (7.5 mg) treatment. Similarly, Murphy et al. [46] also found no behavioural effect on fear recognition of oral citalopram, although the BOLD response to fearful faces in the amygdala was reduced. Harmer et al. [30] and Browning et al. [47] did report enhanced recognition of both fear and happiness in faces by predominantly female healthy volunteers following acute citalopram (oral 20 mg or iv. 10 mg)-though decreased recognition of fear in faces following ATD in female volunteers [48]. It is possible that the effects of SSRIs on emotion are critically dependent on dose and plasma levels; our dose of escitalopram led to plasma levels comparable with the lower end of the clinical range. However, direct comparison with the studies above was not possible as plasma levels were not routinely reported. Of course, it is possible in theory that higher doses of acute escitalopram would have produced more robust effects on emotional processing. Overall however this study shows that behavioural measures of emotional processing are somewhat insensitive to acute SSRI treatment in comparison to more cognitive indices. Indeed, cognitive changes may contribute to emotional sequelae. For example, impaired cognitive flexibility, as shown here, may promote ruminative thinking that leads to anxiety. Such cognitive effects may even contribute to the initial increased anxiety that patients experience when initiating treatment [11].

### Implications for understanding 5-HT function: comparisons with ATD

The inhibitory actions of acute escitalopram administration on terminal 5-HT release, through actions at auto-receptors on raphé 5-HT neurons [8], may be analogous to the presumed transient reduction in 5-HT activity caused by ATD [49]. Nord et al. [50] found that the same dose of escitalopram, as used here (20 mg oral), produced changes in PET ligand binding, suggestive of reduced 5-HT in cortical (rather than subcortical) regions in male humans. Higher acute i.v. doses in female rhesus monkeys produced apparently increased 5-HT levels, particularly in cortical and thalamic regions presumably because auto-receptor effects are outweighed by greater effects at the synaptic terminals.

Acute versus chronic serotonin reuptake inhibition can have opposite functional effects on emotional processing and facets of information processing [6, 51]. Whether these opposite actions are respectively due to globally diminished or enhanced 5-HT activity is still unclear. However, the same acute dose can also produce a mixture of apparently opposite functional effects in different domains, e.g. improved SSRT and worsened ED-shifting, and enhanced processing of both ‘fearful’ and ‘happy’ emotions [47]. This might arise because of differential effects of the SSRI in different brain regions sub-serving these functions, perhaps because of regional marked variations in SERT density [52] or in differences in inhibitory auto-receptors in the dorsal and median raphé nuclei [8], as well as their projections to the forebrain regions (to striatum, amygdala and neocortex and to hippocampus and limbic regions, respectively; [53]). The PET studies by Nord et al. [50] also suggest that they result from diminished 5-HT post-synaptic actions in certain regions, mainly in the neocortex. Alternatively, Del-Ben et al. [39] showed that acute oral citalopram could affect the same orbito-frontal/ventro-lateral PFC region differentially according to the task, as well as producing other region-specific changes in the functional BOLD response.

These observations make it plausible that global changes in 5-HT function produced by escitalopram may have differential effects on cognitive and emotional processes mediated by different brain regions or networks. Another way of formulating this point is that comparable changes in ‘arousal’ produced by alterations in 5-HT function may have differential effects requiring different levels of ‘arousal’ for optimal performance.

Contrasting escitalopram with ATD, the latter may have very different effects on regional 5-HT function, distinct from those produced by acute escitalopram [2]. Although there have been relatively few direct comparisons of these treatments using brain imaging methodology (e.g. [40]), evidently, acute escitalopram and ATD do not necessarily reflect opposite effects on 5-HT functioning.

Primary study limitations were the use of only one dose of escitalopram, although it was the clinically highest allowed one and higher acute doses being precluded by ethical considerations. A sub-chronic study of the effects of this drug is also now urgently indicated to determine to what extent these acute effects persist or even reverse. We employed a between-group design because of well-known practice effects confounding crossover designs on cognitive functions [16]; however, the groups were well matched. We employed a large number of tests of cognition and emotional function, but controlled for false discovery rate across the entire test battery.

### Summary

We showed contrasting detrimental and enhancing effects of acute high-dose escitalopram on cognitive functioning in human volunteers, while probabilistic learning was impaired in a manner consistent with the effects of reduced 5-HT transmission and depression. The results are relevant to understanding the clinical effects of SSRIs at early stages of treatment, as well as the role of 5-HT in emotional and cognitive processing.

## Funding and disclosure

This work was supported by a Wellcome Trust Senior Investigator Award to TW Robbins (104631/Z/14/Z) and the NIHR Cambridge Biomedical Research Centre (Mental Health theme). TWR consults for Cambridge Cognition, Mundipharma and Unilever have held research grants from Shionogi and Lundbeck and receives Royalties for CANTAB. NS was supported by a Medical Research Council Doctoral Grant Studentship (1432057), VV by a Medical Research Council Senior Fellowship (MR/P008747/1), SCR by a Wellcome Trust Clinical Fellowship (110049/Z/15/Z), LP by the Medical Research Council (MR/P01271X/1) and FC is employed by Cambridge Cognition. JBR is supported by the Wellcome Trust (103838) and received research grants unrelated to this work from Medical Research Council, James S McDonnell Foundation, PSPAssociation, AZ-Medimmune, Janssen and Lilly, and served as editor for Brain. RR received a grant (16KNO16238) from the Federal Ministry for Economic Affairs and Energy. BJS consults for Cambridge Cognition, PEAK and Mundipharma and SRC consults for Cambridge Cognition and Shire. JBD, RC and WR Bevan-Jones have nothing for disclosure.

## Electronic supplementary material

Supplementary Materials and Methods
