# Supplementary material for: Dissociable effects of acute SSRI (escitalopram) on executive, learning and emotional functions in healthy humans
Source: Neuropsychopharmacology. 2018 Sep 26;43(13):2645–51. doi: 10.1038/s41386-018-0229-z (PMC6224451; doi:10.1038/s41386-018-0229-z)
Supplement: Supplementary file 1 — Supplementary Materials and Methods [file 41386_2018_229_MOESM1_ESM.docx]

**Supplementary Materials and Methods**

**Study recruitment:** Healthy volunteers aged 18-45 years old were recruited from local community and gave written consent prior to study enrolment. The testing session (starting approximately 9-10 am) was conducted at the NIHR Cambridge Biomedical Research Centre (Mental Health theme) located in Addenbrooke’s Hospital.

**Detailed exclusion criteria:** Participants were screened by a medical doctor for eligibility, which was confirmed with psychiatry specialty registrars. Exclusion criteria were: alcohol abuse (defined as more than 28 UK units per week for males and 21 units per week for females), smoking more than five cigarettes per day, and active or previous medical problems that could affect drug absorption, metabolism and action (including gastrointestinal disorders, cardiac problems and endocrine problems, head injury and epilepsy, impaired vision/hearing/movement, glaucoma, bleeding disorders, asthma, and renal impairment), and any regular medication (apart from the contraceptive pill for females). Participants were requested to report any major illness during the last month prior to study day. Drug abuse was defined, as per Chamberlain et al., 2006, as: 1) use of any listed substances (including cannabis, amphetamines, cocaine, heroin, ecstasy, barbiturates, tranquilizers, opiates or psychedelics) within the last month, 2) more than occasional (i.e. more than five times throughout lifetime) intake of all listed drugs except for cannabis, or, 3) regular (i.e. more than once monthly) cannabis intake. Participants with a personal or family history of cardiovascular (heart or circulation) problems were excluded. Additionally, all participants underwent an ECG to exclude any underlying cardiac abnormalities and female participants underwent a urine test to exclude the possibility of pregnancy. Participants were requested to refrain from alcohol 24 hours prior to and after the end of study day. Prospective participants were also asked if they had participated in another pharmacological study within the last two weeks and if so, they were excluded from study participation.

**Blood sampling:** One participant refused blood sampling and it was unsuccessful for another participant. Overall samples were collected for 64 participants. Blood samples were centrifuged within 20 minutes at 4°C for 10 minutes at 4000rpm by the nursing staff in the National Institute of Health Research/Wellcome Trust Clinical Research Facility in Addenbrooke’s Hospital and plasma was separated and stored at -20°C and subsequently transported in -80°C dry ice for analysis to our collaborator (RR) in Leipzig, Germany. Blood samples for four participants were inappropriately stored due to error and were discarded. Blood samples for one participant not completing neuropsychological testing due to side effects were not included in the analysis.

**Neuropsychological testing:** Following drug administration and prior to testing, participants waited in a quiet room and were offered a light lunch; food does not interact with escitalopram absorption (Rao, 2007). Participants were reimbursed with a fixed amount for the time spent plus a flat amount for travel expenses. Participants were incentivised for optimal task performance by being told they would receive additional monetary reward depending on their performance and thus to try to perform as well as possible. All participants eventually received an additional flat-rate amount. The complete task battery lasted approximately 3.5 hours and participants were offered regular scheduled breaks.

**Participants completing neuropsychological testing**

**Probabilistic learning task:** Data from one participant in escitalopram group were discarded due to technical error.

**CANTAB ID/ED task:** Two participants in the escitalopram group and two in the placebo group did not complete this task.

**Appetitive instrumental learning task and response inhibition task:** All sixty-five participants completed these tasks.

**CANTAB Affective Go/No-Go task:** Five participants (two in the placebo group and three in the escitalopram group) failed to complete this task.

**EMOTICOM Affective Go/No-Go task:** Two participants in the placebo group failed to complete this task.

**EMOTICOM Social information preference task ('Theory of mind'):** Six participants (one in the placebo group and five in the escitalopram group) did not complete this task.

**Data analysis**

Given previously shown differential effects of ATD on emotional processing in male versus female participants (Harmer et al, 2003b) and our sample size being well balanced (overall N=65; N=33 male and N=32 female participants), we included gender in our analyses. As personality traits, including self-reported anxiety (Sen et al, 2004) are associated with the short allele of the 5-HTTLPR polymorphism, we separated participants into high and low trait anxiety groups based on STAI trait anxiety scores to analyse any interaction with escitalopram. Given findings of deficits in attention, learning and memory in depression, we separated participants into low and high BDI groups in the learning and emotional processing tasks.

**
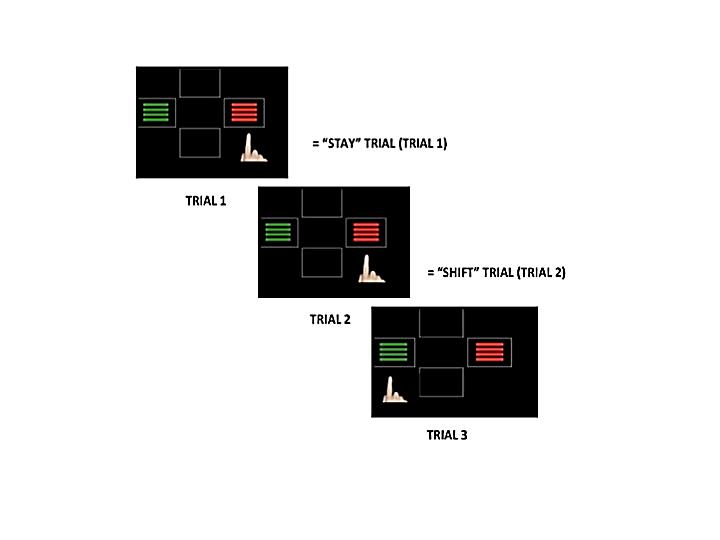
**

**Figure 1: Probabilistic reversal learning task structure.** Participants made a two-alternative forced choice during task and received either accurate (80%) or misleading feedback (20%) presented on the screen after each trial and combined with distinct auditory feedback. Trials were labelled as stay or shift based on whether the participant chose the same pattern or switched in the next trial. For win-stay/lose-shift strategy we calculated the percentage of trials in each condition, i.e. for win-stay condition in stage 1 we divided the number of trials participants stayed after being rewarded, i.e. chose the same stimulus in the next trial, by the total number of trials in this stage

**
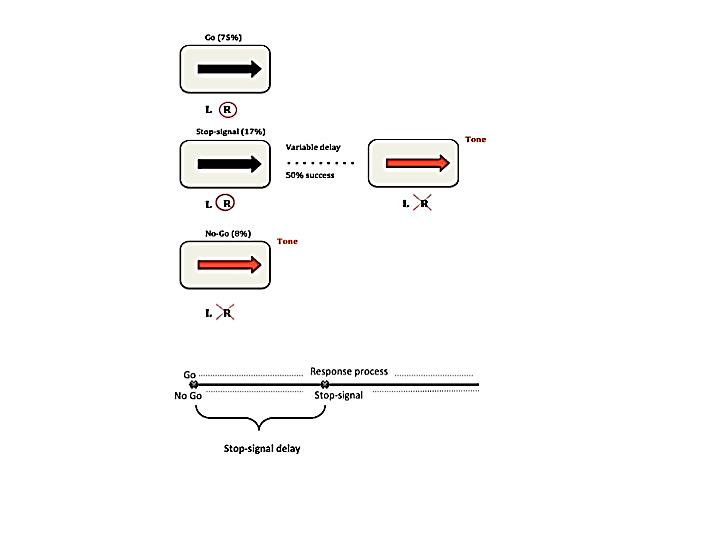
**

**Figure 2:** The interleaved Stop-signal, No-go trials task structure with types and percentage of trials presented to participants. During Go trials, participants see a black arrow and must respond quickly and according to its direction (a left-facing arrow requires a left button press; a right-facing arrow requires a right button press). In Stop-signal trials, the arrow is initially black (indicating that a response is required) but soon afterwards, the arrow turns red and the tone is played, indicating that participants must withhold the response. In No-Go trials, the arrow appears as red instead, and is accompanied by a tone; participants must not respond at all. According to the initial race model (reviewed in Eagle et al, 2008), the probability of inhibiting the response can be changed by altering the time of appearance of the stop-signal during response execution. The Stop-signal trials are more difficult the later the Stop-signal appears. The stop-signal delay (the time between initiation of Go response until the presentation of the stop-signal) varied from trial to trial following a step-up/down algorithm with initial estimate of 250 milliseconds aiming towards maintaining 50% successful inhibition, similar to previous studies (Ye et al, 2014; Chamberlain et al, 2006). Secondary measures included (1) mean reaction time (RT) in correct Go trials, (2) rate of No-Go commission errors which refer to inappropriate button press during No-Go trials, (3) rate of Go commission errors which refer to wrong button press during Go trials and (4) rate of Go omission errors. Mean stop-signal delay did not differ between escitalopram (mean: 289.81, SD: 101.04) and placebo (mean: 258.7, SD: 87.5) groups, p= 0.19. Secondary measures included (1) mean reaction time (RT) in correct Go trials, (2) rate of No-Go commission errors which refer to inappropriate button press during No-Go trials, (3) rate of Go commission errors which refer to wrong button press during Go trials and (4) rate of Go omission errors.

One participant outlier in the SSRT (>2SD from group mean) was excluded. Escitalopram significantly reduced SSRT (speeded response inhibition), p=0.013, t(62)=-2.550, without significant changes in other measures. We explored the analysis in the Stop-signal reaction time measure by winsorizing instead of trimming the outlier and this did not alter the results.


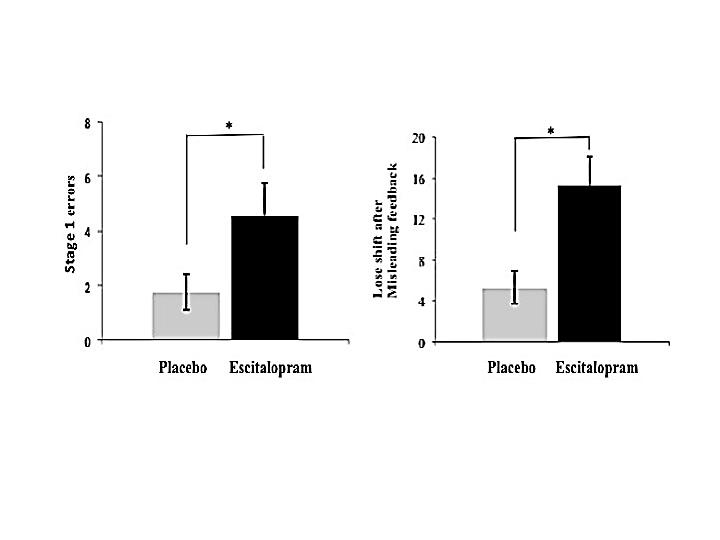


**Figure 3:** Probabilistic learning and reversal task. The escitalopram group made significantly more errors in stage 1 (a) and showed a significantly higher probability to shift after misleading negative feedback (b)

*Significance level set as p< 0.05

**Table 1:** Effects of serotonergic manipulations on the cognitive domains of response inhibition, probabilistic reversal learning and cognitive flexibility

| **Study** | **Task** | **Population** | **Intervention** | **Findings** |
| --- | --- | --- | --- | --- |
| **Response inhibition**  **Action cancellation** | |  |  |  |
| Ye et al., 2014 | Interleaved Stop-signal/No-Go trials | PD patients (21) | Citalopram 30mg | More rapid SSRT and reduced No-Go errors in patients with relatively more severe stage of the disease* |
| Chamberlain et al., 2006 | Stop-signal | HVs (60) | Citalopram 20mg | No effect on SSRT |
| Clark et al., 2005 | Stop-signal | HVs (42) | ATD | No effect on SSRT |
| Bari et al., 2009 | Stop-signal | Rats (26) | Citalopram- various dosages | No effect on SSRT |
| **2. Action restraint** |  |  |  |  |
| Macoveanu et al., 2013 | Go/No-Go | HVs (22) | Citalopram i.v., ATD | No effect on CEs |
|  |  |  |  | Increased BOLD response during No-Go condition 1. in left IFG with citalopram vs. ATD and 2. in right IFG with ATD vs. drug-free |
| Del-Ben et al., 2005 | Go/No-Go | HVs (12) | Citalopram i.v. 7.5mg | No effects on CEs |
|  |  |  |  | Increased right OFC (BA47) activation and decreased medial orbitofrontal activation during No-Go condition |
| Crockett et al., 2009 | Go/No-Go | HVs (22) | ATD | No effect on CEs |
|  |  |  |  | Abolishment of punishment induced slowing of RTs |
| Rubia et al., 2005 | Go/No-Go | HVs (9) | ATD | No effect on CEs |
|  |  |  |  | Reduced BOLD response in right orbito-inferior prefrontal, superior and medial temporal cortex during No-Go |
| **Probabilistic instrumental learning** |  |  |  |  |
| Murphy et al., 2002 | Reversal learning | HVs (11) | ATD | No effect on errors |
|  |  |  |  | Slower RTs |
| Clarke et al., 2004 | Reversal learning | Marmosets (3) | Selective 5,7-DHT induced prefrontal 5HT depletion | Increased perseverative errors after stimulus reversal |
| Clarke et al., 2006 | Serial-discrimination reversal learning | Marmosets (28) | Selective 5,7-DHT induced OFC 5HT depletion | Increased perseverative errors after second stimulus reversal |
| **Cognitive flexibility** |  |  |  |  |
| Rogers et al., 1999 | CANTAB ID/ED | HVs (15) | ATD | Fewer subjects completing stage of CD and more errors in this stage  More errors in IDS |
| Clarke et al., 2005 | CANTAB ID/ED | Marmosets (16) | Selective 5,7-DHT induced prefrontal 5HT depletion | No effect on EDS  Increased perseverative errors after stimulus reversal |

*Abbreviations* **PD:** Parkinson's disease, **CEs:** commission errors, **5,7- DHT:** 5,7-dihydroxytryptamine, **5-HT:** serotonin, **CD:** Compound discrimination, **IDS:** Intra-dimensional set shift, **EDS:** Extra-dimensional set shift**, RTs:** Reaction times**, OFC:** Orbitofrontal cortex, **mCPP:** m-chlorophenylpiperazine**, dmPFC:** dorso-medial prefrontal cortex, **IFG:** inferior frontal gyrus, **SERT:** serotonin transporter, **SSRT:** Stop-signal reaction time**.** A reversal switch error in the probabilistic reversal-learning task denotes an incorrect response where participants reversed on the subsequent trial (Evers et al., 2005). *Quantified with the higher Unified Parkinson’s Disease Rating Scale motor score

**Table 2:** Mean (SD) errors in the learning stages of the CANTAB ID/ED shift task

| **Stages** | **Description** | **Escitalopram group** | **Placebo group** | **Group difference*** |
| --- | --- | --- | --- | --- |
| **1 (SD)** | Simple discrimination | 1.13 (1.61) | 0.79 (0.69) | 0.296 |
| **2 (SR)** | Simple reversal | 2.17 (2.56) | 1.42 (0.58) | 0.197 |
| **3 (C/D)** | Compound discrimination 1 | 0.47(0.9) | 0.64 (0.83) | 0.441 |
| **4 (CD)** | Compound discrimination 2 | 0.3(0.6) | 0.07 (0.26) | 0.067 |
| **5 (CDR)** | Compound reversal | 1.33 (0.61) | 1.25 (.52) | 0.577 |

***Group difference:** p-values following one-way ANOVAs

**Table 3:** Subjective mood ratings presented as a continuous line between two end-points, i.e. alert versus drowsy

| **Subjective mood ratings** | **Placebo**  **Mean (SEM)** |  | **Escitalopram**  **Mean (SEM)** |  |
| --- | --- | --- | --- | --- |
|  | **Baseline** | **Post drug, pre testing** | **Baseline** | **Post drug, pre testing** |
| Alert- drowsy | 35.17 (3.66) | 36.59 (3.63) | 29.72 (4.11) | 43.7 (3.72) |
| Calm- excited | 34.28 (3.63) | 29.07 (3.02) | 30.02 (4.02) | 38.25 (4.11) |
| Strong- feeble | 30.97 (2.17) | 31.78 (3.14) | 27.97 (3.41) | 40.57 (4.42) |
| Muzzy- clear headed | 71.42 (3.13) | 68.57 (2.61) | 72.4 (3.79) | 59.06 (3.7) |
| Well coordinated- clumsy | 23.6 (2.41) | 29.76 (3.09) | 22.57 (3.48) | 39.5 (4.3) |
| Lethargic- energetic | 64.84 (2.98) | 58.99 (3.58) | 64.82 (4.7) | 54.6 (4.2) |
| Contented- discontented | 23.38 (2.58) | 31.40 (3.86) | 25.26 (3.2) | 28.03 (3.1) |
| Troubled- tranquil | 74.64 (2.2) | 70.68 (2.73) | 75.04 (2.81) | 70.01 (3.24) |
| Mentally slow- quick witted | 65.42 (2.32) | 65.30 (3.04) | 67.22 (3.23) | 59.26 (3.89) |
| Tense- relaxed | 76.19 (2.19) | 71.46 (2.69) | 67.59 (3.05) | 66.85 (3.46) |
| Attentive- dreamy | 29.11(2.87) | 36.62 (3.18) | 29.22 (3.22) | 37.68 (3.79) |
| Incompetent- proficient | 73.24 (2.27) | 70.21 (2.39) | 72.84 (2.99) | 62.8 (3.88) |
| Happy- sad | 24.12 (2.23) | 28.51 (3.07) | 25.79 (3.05) | 28.8 (3.73) |
| Antagonistic- friendly | 80.76 (2.16) | 77.10 (2.44) | 79.83 (2.48) | 77.55 (2.7) |
| Interested- bored | 24.76 (2.64) | 32.83 (3.61) | 23.86 (3.22) | 32.76 (3.64) |
| Withdrawn- sociable | 72.58 (2.77) | 65.83 (3.51) | 69.51 (0.6) | 68.6 (4.26) |

Participants had to move the cursor along the line on the screen. Participants in the escitalopram group showed a significant increase in self-reported excitement after drug administration and prior to starting neuropsychological testing p=0.028, F(1,29)=5.368, partial η^2^=0.156 but this disappeared by the end of testing session, p>0.05. There was no other significant difference between treatment groups in other subjective mood ratings during testing session (p> 0.05)
